# Effects of nitrogen fertilizer rates on nutrient uptake, vertical nutrient distribution, and nitrogen balance in dryland spring wheat

**DOI:** 10.3389/fpls.2026.1745172

**Published:** 2026-02-26

**Authors:** Aixia Xu, Khuram Shehzad Khan, Yan Zhang, Nana Liu, Pengbin Liu, Yafei Chen, Xuexue Wei, Chongrui Sun, Zechariah Effah, Lingling Li

**Affiliations:** 1 State Key Laboratory of Aridland Crop Science, Gansu Agricultural University, Lanzhou, China; 2 College of Agronomy, Gansu Agricultural University, Lanzhou, China; 3 Seed Industry Research Institute of Gansu Provincial University, Lanzhou, China; 4 College of Resources and Environmental Sciences, National Academy of Agriculture Green Development, China Agricultural University, Beijing, China; 5 CSIR-Plant Genetic Resources Research Institute (PGRRI), Bunso, Ghana

**Keywords:** N loss, N residual, N surplus, N use efficiency, nutrient uptake

## Abstract

**Introduction:**

Optimizing nitrogen (N) fertilizer management is essential for sustainable crop production in semi-arid, rain-fed agricultural regions. This study evaluated the effects of different N-fertilizer rates on nutrient uptake, soil nutrient distribution, and N balance in spring wheat (*Triticum aestivum L.*) under dryland conditions.

**Methods:**

The analysis was based on a long-term field experiment initiated 2003 in Dingxi, Gansu Province, China, with five N application rates: 0, 52.5, 105.0, 157.5, and 210.0 kg N ha^−1^ (designated as N1-N5).

**Results:**

The study results showed that the concentrations and accumulations of N and K in wheat organs increased significantly with increasing N rates. However, both yield and nutrient uptake plateaued at105 kg N ha^−1^ (N3) and no statistically significant benefits were observed at higher N rates. In contrast, increasing N fertilization significantly reduced grain P concentration, with the N5 treatment showing a 17.80% decrease compared with N1. Soil nutrient responses exhibited clear vertical differentiation. Residual available N in the 0–100 cm soil layer increased significantly with increasing N rates, with a 32.20% increase under N5 compared with N1, whereas available P decreased by 31.49%. Available K showed redistribution characteristics, with the surface layer and enrichment in the subsoil. Nitrogen balance analysis indicated that the apparent N-use efficiency decreased from 95.12% to 39.71% as N rates increased, while the apparent loss rate shifted from negative to positive values, reaching 33.79% under N5. The N3 treatment achieved a near equilibrium N balance (8.69 kg ha^−1^), whereas the N5 treatment resulted in a substantial N surplus of 102.34 kg ha^−1^. Overall, an application rate of 105 kg N ha^−1^ was identified as the optimal N fertilizer rate for dryland spring wheat.

**Discussion:**

This rate ensured adequate N uptake and grain yield, maintained high N use efficiency (NUE), minimized potential environmental risks, and achieved a balanced N supply-demand relationship.

## 1 Introduction

In the context of continuous population growth and intensifying climate change, achieving sustainable agricultural development has become increasingly critical (Godfray et al., 2010; Xu et al., 2025). Among the many factors influencing crop production, nitrogen (N) is a key nutrient limiting crop yield. As a fundamental component of amino acids, proteins, nucleic acids, and chlorophyll, N plays a crucial role in regulating photosynthesis, plant growth, development, and ultimately crop productivity (Hawkesford, 2014; Sinclair and Rufty, 2012). Consequently, the application of chemical N fertilizers has become the most direct and effective strategies for enhancing crop yields in modern agriculture, contributing to the doubling of global grain production since the mid-20th century (Erisman et al., 2008; Guo and Wang, 2021).

N fertilization is often known as “double-edged sword” (Ning et al., 2015). Globally, the seasonal utilization rate of N fertilizer remains low, average 41% (Yan et al., 2025; Zhang et al., 2015). This indicates that more than half of the N fertilizer is not taken up by crops during the current season but remains in the soil-crop system or enters the environment through various pathways, leading to severe ecological and environmental problems (Vuille-dit-Bille et al., 2025; Yan et al., 2025). For example, ammonia volatilization and nitrous oxide (N_2_O) emissions exacerbate air pollution and intensify the greenhouse effect (Kumar et al., 2025; Zhang et al., 2015), while the nitrate leaching leads to groundwater pollution and water eutrophication (Grizzetti et al., 2020). Moreover, N_2_O produced by denitrification is a potent greenhouse gas with a global warming potential far exceeding that of CO_2_ (Gu et al., 2015; Huang et al., 2025). Recently, Zhang et al. (2023) found that the amount of surplus N in the soil of 54 crops in China can be as high as 138–421 kg ha^−1^. The excessive dependence on N fertilizers has significantly boosted food production but has also resulted in severe environmental consequences that surpass the agricultural benefits—a phenomenon referred to by the scientific community as the “N paradox” (Sutton et al., 2011). Therefore, exploring strategies to maximize N use efficiency (NUE) and minimize N losses to the environment, without compromising high and stable crop yields remains a central scientific challenge for achieving sustainable agricultural development.

This challenge is particularly urgent in semi-arid rainfed agricultural regions (Aziz et al., 2025). These regions are typically characterized by low annual precipitation, uneven seasonal rainfall distribution, high evaporation rates, limited irrigation infrastructure, and crop production that is highly dependent on natural precipitation (Aziz et al., 2025; Sutton et al., 2011). The Loess Plateau of China exemplifies these conditions. In this region wheat is the primary staple crop, and its productivity is critical for regional food security. However, poor soil fertility and low organic matter content are major factors limiting crop yield (Stewart and Peterson, 2015). In pursuit of higher productivity, farmers in these regions often apply excessive amounts of N fertilizer. Under rain-fed conditions, however, NUE is constrained by highly variable rainfall, which limits plant N uptake. Moreover, improper N management can intensify N losses through leaching along the soil profile or via surface runoff and volatilization, resulting in inefficient resource use and increased environmental risks (Gao et al., 2017).

Moreover, excessive N fertilization can disrupt the balance of nutrient uptake and translocation within crops, thereby affecting the bioavailability and distribution of other essential nutrients, such as phosphorus (P) and potassium (K) among different plant organs (Liu et al., 2025b). These nutrient imbalances may reduce overall nutrient use efficiency and adversely affect crop quality (Chen et al., 2014; Yu et al., 2025). Therefore, a comprehensive investigation of the fate of N and its interaction with other nutrients in the wheat-soil system in semi-arid, rainfed regions is of both theoretical and practical importance for optimizing fertilizer management strategies and minimizing environmental risks.

The uptake and internal distribution of nutrients by crops are crucial physiological processes linking soil fertility to crop yield (Ding et al., 2025). Nitrogen application rates strongly influence the concentration and accumulation of N, P, and K in different crop organs (Ding et al., 2025). Previous studies have demonstrated that appropriately increasing N-fertilizer application can enhance N uptake, increase leaf chlorophyll content and photosynthetic capacity, and ultimately improve biomass production and grain yield (Dordas, 2009; Xu et al., 2024). Additionally, N regulates the uptake and transport of P and K. For instance, insufficient N supply restricts root growth, thereby indirectly reducing the ability to absorb P and K. Conversely, excessive N fertilization may lead to “luxury uptake” of N by crops, resulting in nutrient imbalances, such as inhibiting P transport to grains or altering K distribution between vegetative and reproductive organs (Cameron et al., 2013; Gaju et al., 2014). The “synergistic” and “antagonistic” effects of nutrients ultimately determine crop yield and nutrient use efficiency. However, most studies focus solely on N or on a single crop organ (such as grain). There is a lack of systematic research on the dynamic distribution of N, P, and K across the entire plant (leaves, stems, chaff, and grains) from anthesis to maturity, as well as their relative contribution to yield formation under different N rates, particularly in semi-arid environments.

Soil serves as central medium for nutrient cycling, and fertilization exerts a significant impact on the soil nutrient pool. The fate of N fertilization includes crop uptake, soil retention (mainly as inorganic N in various soil layers), and losses through various pathways (Yan et al., 2025). In rain-fed agricultural areas, the residual and mobility of soil inorganic N (especially NO_3_-N) are substantial, and its vertical distribution across soil layers is a critical index for assessing leaching risk (Ju et al., 2006; Zhang et al., 2025). High N input often results in NO_3_-N leaching into deeper soil layers, forming a potential “chemical time bomb” (Sun et al., 2024). Long-term N fertilization significantly alters the form and availability of soil P and K (Ladha et al., 2016). Studies indicate that N fertilizer-induced acidification can activate certain forms of P. However, it may also increase P fixation in soil, reducing its effectiveness through ion competition or alterations in microbial activity (Guo et al., 2010; Römheld and Kirkby, 2010). For K, N fertilizer can affect its adsorption-desorption equilibrium on soil colloids and its distribution across soil layers (Sainju, 2017). Systematic elucidation of the response and vertical differentiation of soil N, P, and K nutrient pools under varying N levels, is essential for comprehensively evaluating the environmental impacts of N fertilization and guiding nutrient management strategies.

N balance and its N input-output ratio in farmland systems are crucial indicators for evaluating the rational N rate and their environmental impact (Huang et al., 2020; Sainju et al., 2016). By calculating apparent equilibrium, key efficiency indicators such as apparent utilization rate, residual rate, and loss rate of N can be estimated (Zhang et al., 2015). Among these, the apparent surplus of N serves as a comprehensive index. When it is close to zero, it suggests that the supply and demand of N in the system are roughly balanced (Yu et al., 2025); a significant surplus indicates a higher environmental risk (Zhang et al., 2023). Conversely, a deficit suggests the depletion of the soil N pool, which is detrimental to the sustainable development of farmland systems (Ji et al., 2017). Numerous studies have employed this method to evaluate the effects of N management in various farmland ecosystems (Zhao et al., 2010). However, in semi-arid rain-fed areas with significant interannual precipitation variability, N balance research based on long-term positioning experiments can more accurately reflect the N cycle. Such studies provide robust scientific basis for determining environmentally appropriate N application rates and improving nutrient management in these fragile ecosystems.

In summary, determining an optimal N application rate that balances high crop yield, efficient nutrient utilization, and environmental protection remains a major challenge in semi-arid, rain-fed agricultural regions. This study is based on a long-term N rates experiment initiated in 2003 in Dingxi City, Gansu Province, focusing on the spring wheat soil system. Five N rates (0, 52.5, 105.0, 157.5, and 210 kg N ha^−1^) were tested. The core objectives of this study were to: (i) elucidate the concentration and accumulation patterns of N, P, and K in different wheat organs, examine their translocation dynamics from anthesis to maturity, and analyze their relationship with grain yield; (ii) characterize the vertical distribution and residual dynamics of total and available N, P, and K in the 0–200 cm soil profile under different N fertilization; and (iii) quantify the fate and apparent balance of N in the topsoil by calculating N apparent surplus amount (NASA), N apparent residual rate (NARR), N apparent utilization efficiency (NAUE), and N apparent loss rate (NALA). Through this study, we aim to clarify the key processes governing N regulation and nutrient cycling in the wheat soil system under semi-arid, rain-fed conditions. These findings provide both a theoretical basis and empirical support for developing high-yield, resource-efficient, and environmentally sustainable N management strategies for this region and similar dryland agroecosystems.

## 2 Materials and methods

### 2.1 Experimental site

**Figure 1:**
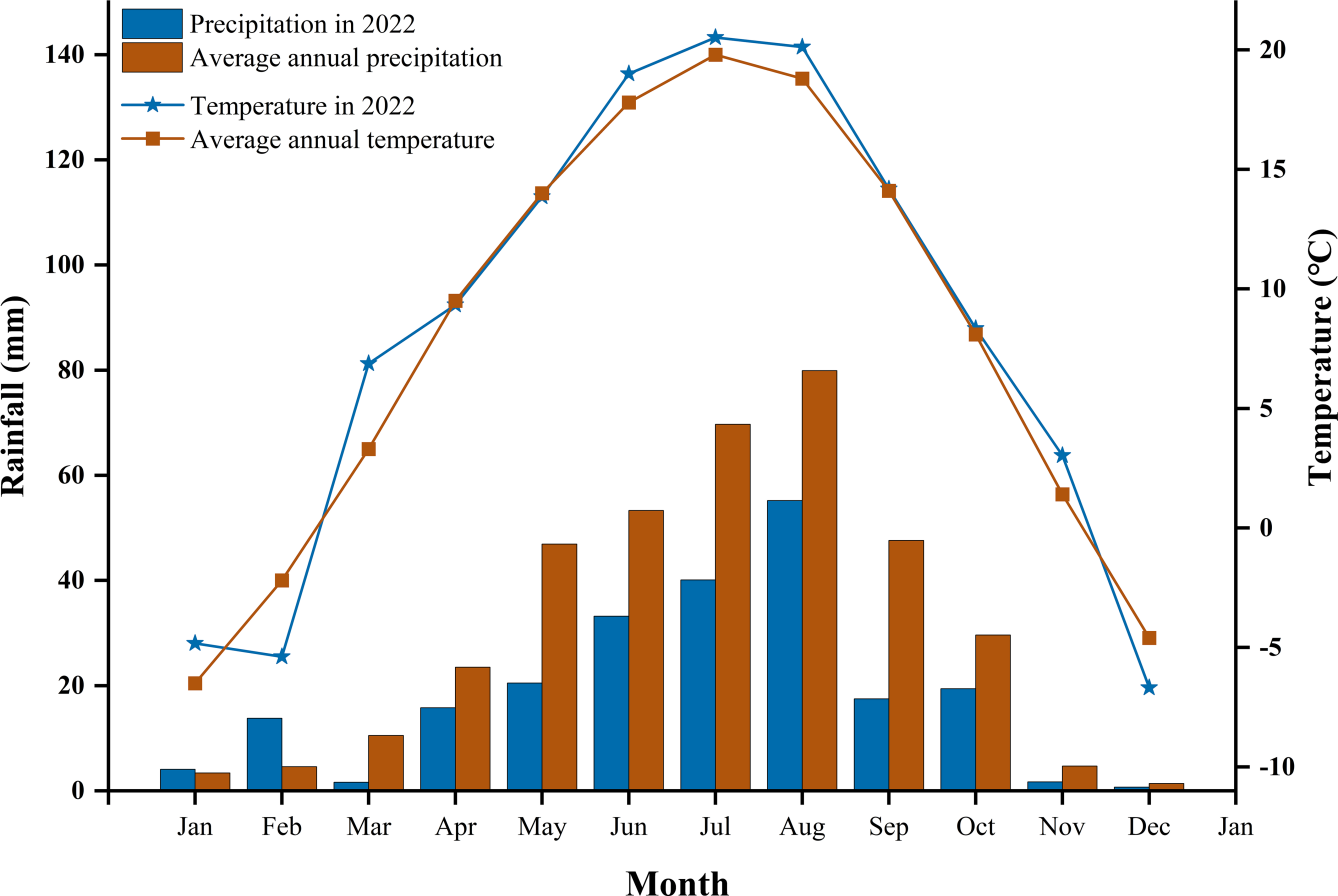
Rainfall and temperature in 2022 and the average from 2003 to 2022.

The research site is located in the Anding District of Dingxi City, within the hilly terrain of central and southern Gansu Province. This area typifies a semi-arid, rain-fed agricultural zone without irrigation systems. Since 2003, an extensive study on N fertilization in spring wheat has been conducted here. From 2003 to 2022, temperatures from January to July ranged between -22°C and 38°C (**Figure 1**). The mean annual temperature was 6.4 °C, with accumulated temperatures ≥10 °C totaling 2240 °C. The annual mean precipitation was 390.7 mm, with an evaporation rate of 1531 mm and a coefficient of variation for precipitation of 24.3%. The average annual solar radiation reached 5930 MJ m^−2^, with a total sunshine duration of 2480 hours. The frost-free period lasted approximately 140 days. Rainfall in 2022 was 223.6 mm, and the average temperature was 8.3 °C. The area has a rich agricultural history, initially centered on flaxseed (*Linum usitatissimum* L.) cultivation. The primary physical and chemical properties of the 0–200 cm soil profile are as follows: TN 0.73 g kg^−1^, NH_4_-N 4.90 mg kg^−1^, NO_3_-N 25.68 mg kg^−1^, pH 8.36, TP 1.77 g kg^−1^, AP 4.60 mg kg^−1^, TK 18.32 g kg^−1^, AK 169.13 mg kg^−1^, soil bulk density 1.18 g cm^−3^, and organic matter content 11.86 g kg^−1^.

### 2.2 Experimental design

The study employed a completely randomized block design with three replications, each covering an area of 30 m^2^. Five nitrogen (N) fertilizer treatments (N1-N5) were applied at annual rates of 0, 52.5, 105.0, 157.5, and 210.0 kg N ha^−1^, respectively. Urea (46% N) was used as the N fertilizer, and all treatments received 105 kg P_2_O_5_ ha^−1^ as superphosphate (16% P_2_O_5_). All fertilizers were evenly broadcast across the plots in a single application on the day of sowing and subsequently incorporated into the 0–20 cm soil layer using a rotary tiller. Dingxi 40, a high-yielding spring wheat variety, was sown in mid-March each year at a seeding rate of 187.5 kg ha⁻^2^ with a row spacing of 20 cm. Harvesting occurred from late July to early August. Manual weeding was performed during the growing season, and Roundup^®^ (glyphosate, 10%) was applied as needed during the fallow period after harvest. Comprehensive pest management followed local agronomic guidelines. These procedures remained consistent throughout the study period from 2003 to 2022.

### 2.3 Sampling and analysis

At anthesis and maturity, all wheat within a 1 m × 1 m area was harvested and collected. At anthesis, plants were separated into three components: leaves, stems, and ears. At maturity, they were divided into four components: leaves, stems, chaff, and grain. The crop samples were dried at 70 °C until reaching a constant weight. Each crop sample was ground to a size of < 2 mm for nutrient analysis. Crop N, P, and K concentrations were determined using Nessler colorimetry, molybdenum yellow colorimetry, and flame photometry, respectively.

Soil samples were collected annually from each plot using a 4.0 cm inner-diameter auger at depths of 0–5, 5–10, 10–30, 30–50, 50–80, 80–110, 110–140, 140–170, and 170–200 cm, both before and after the wheat harvest each year. For each soil layer within a plot, three cores were combined to create one composite sample, which was subsequently divided into two sub-samples. One sub-sample was immediately transported to the laboratory on ice and stored at -20 °C for soil NO_3_-N analysis, while the other was air-dried, ground to pass through a 2 mm sieve, and used for the analysis of soil chemical properties. Soil total nitrogen (TN) was determined using the standard semi-micro Kjeldahl method. Soil NO_3_-N and NH_4_-N were determined by spectrophotometry. Total phosphorus (TP) and available phosphorus (AP) in the soil were measured using standard molybdenum-antimony colorimetry. The total potassium (TK) in the soil was determined using the NaOH melting flame photometric method, and the available potassium (AK) was assessed using the 1.0 M CH_3_COONH_4_ extraction method (Bao, 1999).

### 2.4 Calculation

The apparent N balance is defined as the difference between N input and N output. In this study, soil N input and output were calculated based on the principle of apparent N balance, excluding nutrients from seeds, precipitation, and atmospheric deposition. The N input was categorized into fertilizer N, soil inorganic N before sowing, and net N mineralization. The N output, excluding losses due to leaching, volatilization, and reverse digestion, was divided into crop N uptake and soil inorganic N residual. Soil inorganic N accumulation (N_IN_, kg ha^−1^), N apparent surplus amount (NASA, kg ha^−1^), N apparent residual rate (NARR, %), N apparent utilization efficiency (NAUE, %), and N apparent loss rate (NALA, %)were calculated using (Equations 1−6) (Ju et al., 2006; Pan et al., 2017; Sainju et al., 2016; Sainju, 2017).

(1) $${\text{N}}_{\text{IN}}\;=\;\sum_{i=1}^{n}\frac{{\text{S}}_{\text{T}}\times {\text{S}}_{\text{BD}}\times {\text{N}}_{\text{C}}}{10}$$

(2) $$\text{NASA }={\text{ N}}_{\text{in}}{\text{+ N}}_{\text{f}}-{\text{N}}_{\text{res}}-{\text{N}}_{\text{up}}$$

(3) $$\text{NARR }=\;\frac{{\text{N}}_{\text{resN}}-{\text{N}}_{res0}}{{\text{N}}_{\text{f}}}\;\times \;100$$

(4) $$\text{NAUE }=\;\frac{{\text{N}}_{\text{upN}}-{\text{N}}_{up0}}{{\text{N}}_{\text{f}}}\times 100$$

(5) NALA = 100 −NAUE−NARR

(6) $$\text{NNM }={\text{ N}}_{up0}+{\text{N}}_{in0}-{\text{N}}_{res0}$$

Where S_T_ (cm) represents soil layer thickness, S_BD_ (g cm^−3^) denotes the soil bulk density, and N_C_ indicates the concentration of NO_3_-N and NH_4_-N (mg kg^−1^) in various soil layers. The variable *n* represents the soil layer. N_in_ (kg ha^−1^) denotes the N_IN_ before sowing, N_f_ (kg ha^−1^) is the N-fertilizer rate, N_res_ denotes the N_IN_ post-harvest, and N_up_ (kg ha^−1^) signifies aboveground crop N uptake. N_in0_ (kg ha^−1^) denotes the N_IN_ before sowing in non-N plot. N_resN_ and N_res0_ represent the N_IN_ post-harvest in N-fertilized and non-N plots, respectively. Similarly, N_upN_ and N_up0_ (kg ha^−1^) represent the aboveground crop N uptake in N-fertilized and non-N plots, respectively.

### 2.5 Statistical analysis

Data analysis was conducted using IBM SPSS software (version 23.0; SPSS Inc., Chicago, IL, USA). The impact of N-fertilizer rates on crop nutrient concentration, soil nutrient concentration, crop nutrient uptake, soil nutrient residue, and N-use efficiency was evaluated through one-way analysis of variance (ANOVA). Significant differences between treatments were determined using Duncan’s multiple range test (DMRT) at a significance level of *p* < 0.05. Pearson correlation coefficients were calculated to assess linear relationships among the variables. Partial least squares regression (PLS) was used to analyze the relationship between crop nutrient concentration, N rate, and grain yield. The results were visualized using SigmaPlot 12.5 and Origin 2021 software.

## 3 Results

### 3.1 Crop nutrient concentration

#### 3.1.1 Crop N concentration

**Figure 2:**
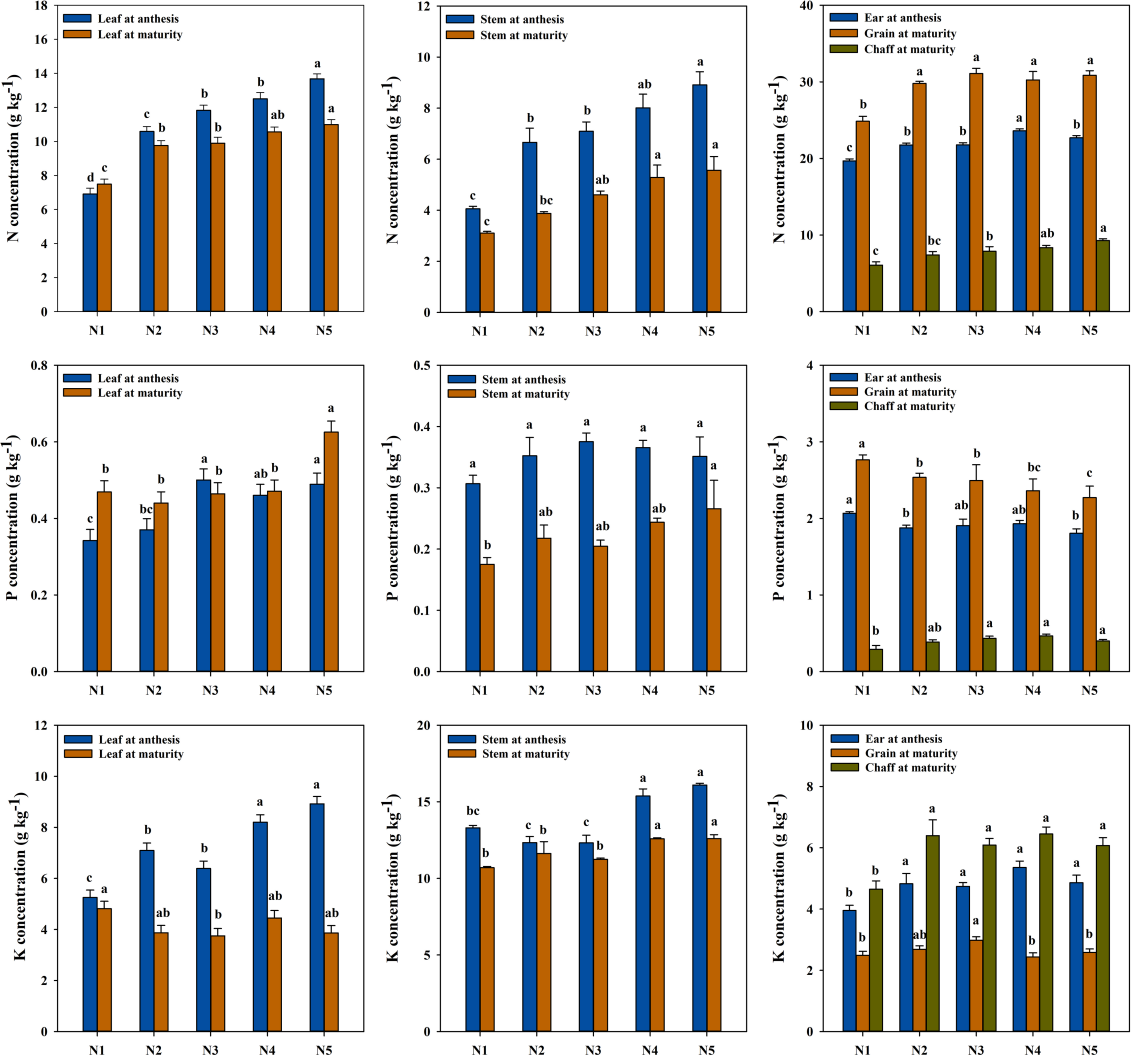
Concentrations of N, P, and K in wheat at different N fertilizer rates: N1, non−fertilized control; N2–N5, annual application of 52.5, 105.0, 157.5, and 210.0 kg N ha^−1^, respectively (2003–2022). Error bars indicate the standard deviation. Bars with different letters indicate significant differences between treatments (*p* < 0.05).

Nitrogen (N) concentrations in all wheat organs at anthesis significantly increased with higher N-fertilizer rates (**Figures 2A–C**). Leaf N concentration rose from 6.91 g kg^−1^ in the non-N control (N1) to 13.68 g kg^−1^ in the N5 treatment, representing an increase of 97.94%, with significant differences observed among N-treatments. Stem N concentration reached a maximum of 8.91 g kg^−1^ in the N5 treatment, 119.54% higher than in the N1 treatment (*p* < 0.05). Ear N concentration peaked at 23.62 g kg^−1^ in the N4 treatment but slightly decreased to 22.70 g kg^−1^ in the N5 treatment. Overall, the N concentration in whole wheat increased by 27.21%, 32.77%, 43.97%, and 47.70% as the N rate increased. However, when the N rate exceeded 157.5 kg N ha^−1^, the increase in N concentration was no longer significant.

At maturity, N concentrations in various organs followed the order: grain > leaf > chaff > stem. N application significantly increased the grain N concentration; however, no statistically significant benefits were observed among the N application treatments (N2−N5). Compared to N1, the N5 treatment resulted in increases of 46.64%, 79.21%, and 52.82%, respectively. The overall N concentration in whole wheat increased by 22.39%, 28.77%, 31.13%, and 36.55% as N-fertilizer rates increased. Beyond 105 kg N ha^−1^, further increases in N concentration were not significant.

From anthesis to maturity, N transport efficiency to grain did not continuously increase with higher N-fertilizer rates but peaked at 42.74% in the N3 treatment. Concurrently, leaf N concentration decreased by 16.32%. Under the N5 treatment, leaf N concentration at maturity remained high at 10.99 g kg^−1^, a 19.7% decrease from anthesis but significantly less than the 7.8% decrease observed under the N2 treatment.

#### 3.1.2 Crop P concentration

Phosphorus (P) concentration in leaves at anthesis showed a significant upward trend with increased N-fertilizer rates (**Figures 2D–F**). Compared to N1, the leaf P concentration increased significantly by 46.15% and 42.94% under the N3 and N5 treatments, respectively. In contrast, the stem P concentration remained relatively stable across treatments, ranging from 0.31 to 0.38 g kg^−1^. Ear P concentration followed an opposite trend, being highest in the N1 treatment (2.07 g kg^−1^), while significantly decreasing to 1.81 g kg^−1^ in the N5 treatment (12.67% decrease, p < 0.05). At the whole-plant level, the P concentration remained consistent (0.87-0.93 g kg^−1^) with no statistically significant benefits observed from the treatments.

At maturity, P concentrations followed the order: grain > leaf > chaff > stem. Grain P concentration peaked at 2.77 g kg^−1^ in the N1 treatment, significantly higher than 2.27 g kg^−1^ in the N5 treatment (17.80% decrease). Leaf P concentration in the N5 treatment increased significantly to 0.63 g kg^−1^, 33.24% higher than in the N1 treatment (p < 0.05). P concentration in the chaff increased with higher N-fertilizer rates, reaching 0.47 g kg^−1^ in the N4 treatment, 60.71% higher than in the N1 treatment (p < 0.01). At the whole-plant level, the P concentration also remained consistent (0.89-0.92 g kg^−1^) with no statistically significant benefits observed from the treatments.

Compared to anthesis and maturity, P distribution exhibited significant spatio-temporal heterogeneity. The transfer rate of P from ears to mature grain ranged from 22.27% to 35.21%. P concentration in the N3 leaves decreased by 7.20%, while other treatments showed increases of 2.39% to 37.10%. Notably, grain P concentration in the N5 treatment was significantly lower than in other treatments (p < 0.05), but leaf P concentration was the highest (0.63 g kg^−1^).

#### 3.1.3 Crop K concentration

Potassium (K) concentration in leaves at anthesis significantly increased with higher N rates (**Figures 2G–I**). The leaf K concentration in the N5 treatment was the highest, 69.79% higher than that in the N1 treatment (p < 0.01). The stem K concentration peaked at 16.09 g kg^−1^ in the N5 treatment, 21.00% higher than in the N1 treatment (p < 0.05). However, the ear K concentration did not show any statistically significant benefits among the N treatments. The whole plant K concentration in the high N treatments (N4 and N5) increased significantly by 28.56% and 32.74%, respectively, compared to the N1 treatment.

At maturity, the K concentration followed the order: stem > chaff > leaf > grain. The grain K concentration was highest in the N3 treatment, significantly higher than in the N1 and N5 treatments. Leaf K concentration generally decreased at maturity, with the N3 treatment showing the lowest level, significantly lower than the N1 treatment (a decrease of 22.17%, p < 0.05), indicating that N supply promoted the redistribution of K to non-grain organs after anthesis. However, the chaff K concentration did not show any statistically significant benefits among the N treatments. The whole plant K concentration was highest in the N4 treatment (6.48 g kg^−1^), but did not increase further in the N5 treatment (6.28 g kg^−1^). Notably, the grain K concentration in the N5 treatment (2.58 g kg^−1^) was significantly lower than in the N3 treatment (p < 0.05), while the stem K concentration (12.6 g kg^−1^) remained high.

#### 3.1.4 Relationship between crop nutrient concentration and grain yield

**Figure 3:**
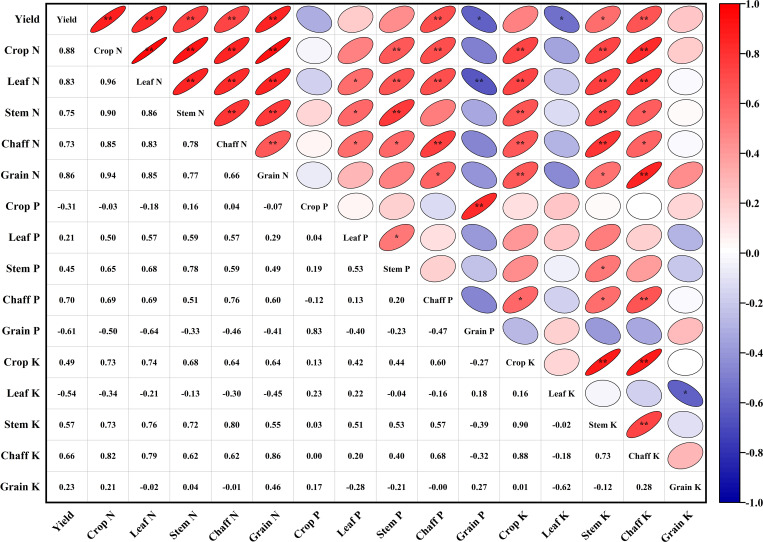
Relationship between crop nutrient concentration (g kg^−1^) and grain yield (kg ha^−1^). * *p* < 0.05; ** *p* < 0.01.

The N concentration showed a significant positive correlation with grain yield at maturity (p < 0.01; **Figure 3**). In contrast, the relationship between P concentration and yield varied across plant organ. While whole-plant P concentration showed no correlation with yield, grain P concentration was negatively correlated (p < 0.05), whereas shell P concentration demonstrated a positive correlation (p < 0.01). Similarly, the correlation between K concentration and yield also differed among plant organs. No significant correlations were observed for whole-plant or grain K concentrations. However, shell K concentration was positively correlated with yield (p < 0.01), while leaf K concentration at maturity showed a significant negative correlation (p < 0.05).

**Figure 4:**
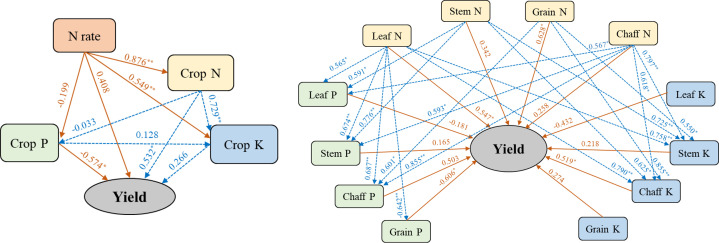
Partial Least Squares Regression (PLS) analysis of crop nutrient concentration (g kg^−1^), N rate, and grain yield under different N-fertilizer rates. Solid orange unidirectional arrows, labeled with orange numbers, denote direct relationships and their corresponding regression coefficients; dotted blue unidirectional arrows, labeled with blue numbers, indicate indirect relationships and their respective regression coefficients. * and ** signify statistical significance at *p* < 0.05 and *p* < 0.01, respectively.

The N-fertilizer rate did not have a significant direct positive effect on yield (0.408, p > 0.05) (**Figure 4**). However, it exhibited a significant indirect positive effect on yield by significantly increasing crop N concentration (direct effect 0.876) and K concentration (indirect effect 0.729). From an organ-specific perspective, the N-fertilizer rate positively influenced yield primarily by enhancing N concentration in leaves (direct effect 0.547) and grains (direct effect 0.628). Additionally, the increased N concentration in leaves (0.790) and grains (0.855) significantly increased chaff K, which subsequently had a significant indirect positive effect on yield (0.519). The N rate did not show a significant direct effect on crop P concentration (-0.199, p > 0.05). Nevertheless, the rise in leaf N concentration significantly increased grain P concentration (0.642), which in turn had a significant indirect negative effect on yield (-0.606).

### 3.2 Soil nutrient concentration after harvest

#### 3.2.1 Soil N concentration

**Figure 5:**
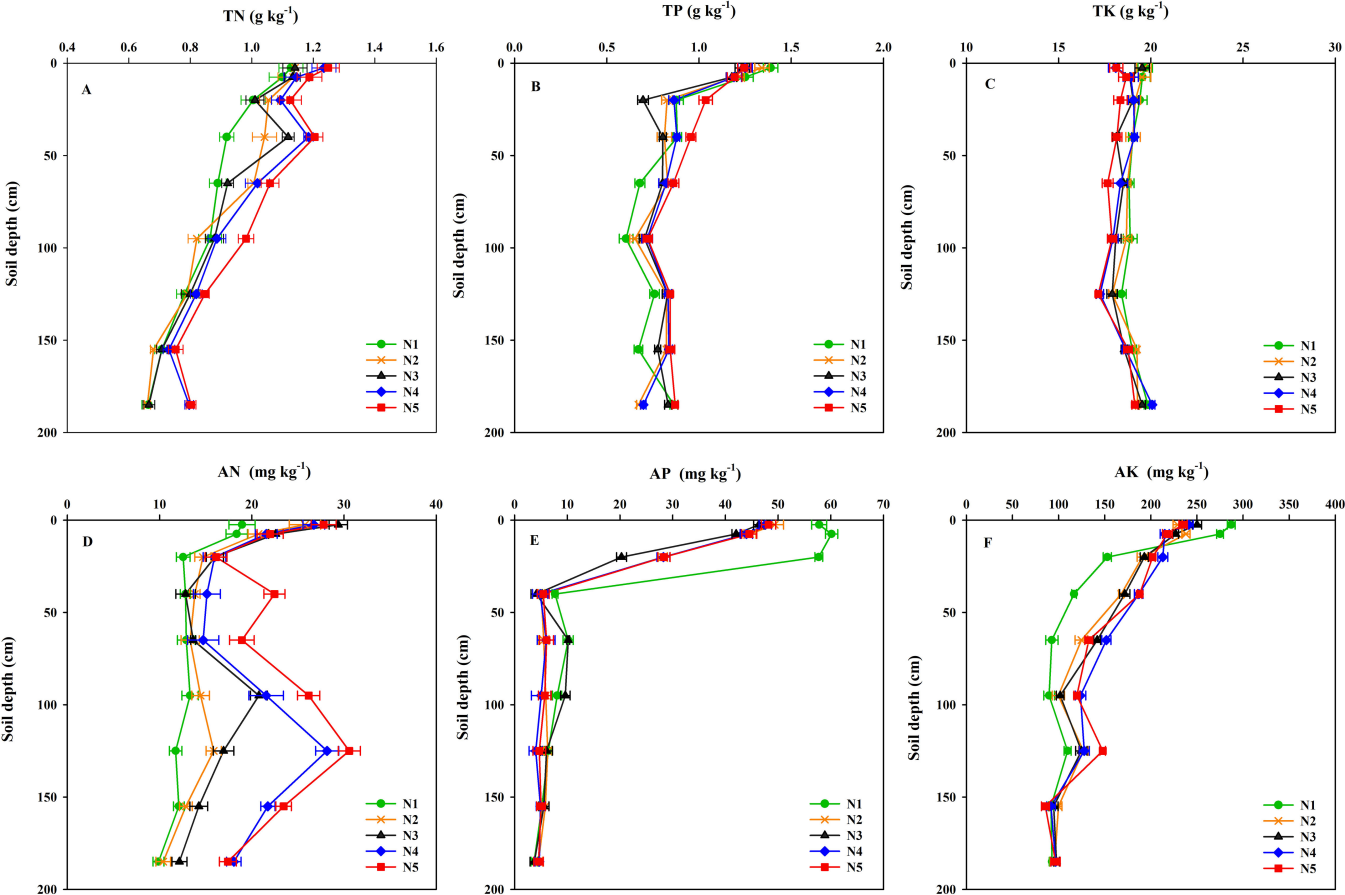
Nutrient concentrations in the 0−200 cm soil layers at wheat maturity: **(A)** Total nitrogen (TN, g kg^−1^), **(B)** Total phosphorus (TP, g kg^−1^), **(C)** Total potassium (TK, g kg^−1^), **(D)** Inorganic nitrogen (AN, mg kg^−1^), **(E)** Available phosphorus (AP, mg kg^−1^), and **(F)** Available potassium (AK, mg kg^−1^). N-fertilizer rates were: N1, non−fertilized control; N2–N5, annual application of 52.5, 105.0, 157.5, and 210.0 kg N ha^−1^, respectively (2003–2022). Error bars indicate the standard deviation.

The N-fertilizer rate significantly impacted the total nitrogen (TN) concentration in the surface soil (0–50 cm) but had minimal effect on deeper layers (> 50 cm) (**Figure 5A**). N markedly accumulated in the surface layer. As the N rate increased from 0–210 kg ha^−1^, the TN concentration in the 0–5 cm layer significantly rose from 1.13 g kg^−1^ (N1) to 1.25 g kg^−1^ (N5, *p* = 0.034). Under the high N treatment (N5), the TN concentration increased by 10.73% and 9.47% compared to the N1 and N2 treatments, respectively. In the 30–50 cm soil layer, the TN concentration in the N5 treatment reached 1.20 g kg^−1^ (*p* < 0.001), significantly higher than in the low N (N2) and non-N (N1) treatments, with increases of 31.14% and 15.65%, respectively. In deeper soil layers (> 80 cm), the effect of N rate varied. The TN concentration in the 80–110 cm soil layer significantly increased to 0.98 g kg^−1^ (*p* = 0.032) under the N5 treatment, while no significant changes were observed in the 110–140 cm and 140–170 cm soil layers. Notably, in the 170–200 cm soil layer, the TN concentration significantly increased to 0.80 g kg^−1^ (*p* = 0.007) under the N4 and N5 treatments, with increases of 24.85% and 29.82% compared to the N1 treatment, and increases of 19.69% and 20.59% compared to the medium N treatment (N3). Although the increase in N rate generally elevated the TN concentration across all soil layers, the response intensity varied significantly among different layers.

The effect of different N-fertilizer rates on the soil inorganic nitrogen (AN) concentration was significant (*p* < 0.05) (**Figure 5D**). With the increase in N rate, the vertical distribution of soil AN exhibited distinct stratification. In the topsoil (0–30 cm), the AN concentration initially increased and then slightly decreased with higher N-fertilizer rates in the 0–5 cm soil layer. The N3 treatment (105 kg N ha^−1^) reached the highest value (29.38 mg kg^−1^), which was 55.11% higher than N1 (*p* < 0.001). In the 5–10 cm and 10–30 cm soil layers, the AN concentration in the N3-N5 treatments increased by 17.46-28.38% compared to N1 (*p* < 0.05). In the middle soil layer (30–80 cm), the AN concentration in the N5 treatment was significantly the highest (22.45 mg kg^−1^) in the 30–50 cm soil layer, which was 75.40% higher than in N1 (*p* < 0.001). In the 50–80 cm soil layer, the AN concentration in the N5 treatment increased significantly by 46.79% compared to N1 (*p* < 0.001). In the deep soil (80–200 cm): from 80–110 cm to 170–200 cm soil layers, the AN concentration in the N4 and N5 treatments was significantly higher than in other treatments (*p* < 0.001). In the 170–200 cm soil layer, N4 (18.06 mg kg^−1^) and N5 (17.4 mg kg^−1^) increased by 82.72% and 76.12%, respectively, compared to N1 (9.88 mg kg^−1^).

#### 3.2.2 Soil P concentration

The effect of N rate on total phosphorus (TP) concentration in soil layers varied significantly (**Figure 5B**). In the 0–5 cm layer, TP concentration under the N1 treatment was significantly higher than N3, N4, and N5 by 11.09%, 9.94%, and 10.30%, respectively. No significant differences in TP concentration were observed among treatments in the 5–10 cm layer (*p* = 0.481). Under the 10–30 cm soil layer, the high N treatment (N5, 210 kg N ha^−1^) significantly increased TP concentration to 1.04 g kg^−1^ (*p* < 0.001), representing an 18.26% increase compared to N1 (0.88 g kg^−1^). Similarly, under the 30–50 cm soil layer, TP concentration peaked under N5 at 0.96 g kg^−1^ (*p* = 0.023). Further, Under the 50–80 cm layer, N rate significantly affected TP distribution (*p* = 0.011), with N5 (0.86 g kg^−1^) exceeding N1 (0.68 g kg^−1^) by 26.79%. In soil layers below 80 cm, TP concentration showed minimal response to N rate.

The N rate significantly affected the available phosphorus (AP) concentration across different soil layers (*p* < 0.05) (**Figure 5E**). As the N rate increased, the AP concentration in the surface to middle soil layers generally decreased. However, at higher N-fertilizer rates, some soil layers exhibited an increasing trend, whereas deep soil layers were less affected. In the topsoil (0–30 cm), particularly in the 0–5 cm layer, an increased N rate led to a significant decrease in AP concentration by 14.60-19.80%, with the N3 treatment reaching the lowest value (46.36 mg kg^−1^). The N4 and N5 treatments showed slight increases (47.57 and 48.21 mg kg^−1^, respectively). Similar trends were observed in the 5–10 cm and 10–30 cm soil layers. In the middle soil layer (30–80 cm), particularly in the 30–50 cm layer, the AP concentration under the N1 treatment was the highest (7.65 mg kg^−1^), which significantly decreased with increasing N rate, with the N3 treatment (4.15 mg kg^−1^) reaching the lowest value, 45.80% lower than under the N1 treatment (*p* < 0.001). In the 50–80 cm soil layer, the AP concentration under the N3 treatment (10.16 mg kg^−1^) was not significantly different from that under the N1 treatment, but the N2, N4, and N5 treatments significantly decreased (5.53, 5.98, and 6.05 mg kg^−1^, respectively). In deep soil layers (80–200 cm), particularly in the 80–110 cm layer, the high N treatments (N4 and N5) significantly decreased the AP concentration by 37.62% and 29.18% compared with the N1 treatment. In the 110–140 cm soil layer, the AP concentrations under the N4 and N5 treatments were significantly reduced by 37.29% and 26.78%, respectively, compared with the N1 treatment. Notably, the N3 treatment had the highest AP concentration in the 50–110 cm soil layer. In the 140–200 cm soil layer, the effect of N rate was diminished (*p* > 0.05).

#### 3.2.3 Soil K concentration

The N application rate significantly influenced the total potassium (TK) concentration in surface soil layers, while its effect on deeper soil layers was minimal (**Figure 5C**). In the 0–5 cm layer, TK concentration levels under high-N treatments (N4 and N5) were significantly lower than those under other treatments, with reductions of 8.12% and 7.97%, respectively, compared to N1. No significant differences were observed among treatments in the 5–10 cm layer (*p* = 0.184). However, in the 10–30 cm soil layer, the TK concentration under N1 (19.4 g kg^−1^) was 5.41% higher than that under N5 (*p* = 0.008). In deeper soil layers (50–200 cm), TK concentrations exhibited only a weak response to variations in N application rates.

The N rate significantly reduced the concentration of available potassium (AK) in the surface layer but promoted its accumulation in the middle layer (**Figure 5F**). In the 0–5 cm soil layer, the AK concentration in N1 was significantly higher than in the other N treatments (*p* < 0.001). Increasing the N rate led to a decrease in AK concentration by 12.92-20.25%, a trend also observed in the 5–10 cm soil layer. However, in the 10–30 cm soil layer, higher N-fertilizer rates significantly increased the concentration of AK. The maximum concentration of AK in the high N treatments (N4 and N5) increased by 39.35% and 32.11%, respectively, compared to N1, while the low N (N2) and medium N (N3) treatments increased by 24.92% and 26.23%, respectively, compared to N1. This indicates that the N rate promoted the migration or release of K to the middle soil. In the 30–50 cm soil layer, the concentration of AK in the N4 and N5 treatments increased by 60.10% and 60.96%, respectively, compared to N1 (*p* < 0.001). In the 50–80 cm soil layer, N4 and N5 increased by 63.80% and 43.26%, respectively, compared to N1 (*p* < 0.001). The response of AK in deep soil (110–200 cm) to N rate was weak (*p* = 0.314-0.943). Only in the 80–110 cm soil layer did N4 (123.77 mg kg^−1^) and N5 (119.76 mg kg^−1^) show significant increases of 38.00% and 33.53%, respectively, compared to N1. Below 140 cm, the AK concentration remained stable (85.68-99.83 mg kg^−1^) with no significant differences among treatments.

### 3.3 Nutrient partitioning and N balance

#### 3.3.1 Crop nutrient uptake

The N rate significantly increased the N content of various wheat organs (**Table 1**). The response intensity varied among organs. Grain N accumulation was the most sensitive to the N rate (*p* = 0.001), followed by the stem (*p* = 0.002) and chaff (*p* = 0.004). The leaf response was also significant (*p* < 0.001), but its increase plateaued at higher N levels. As the N rate increased from 0 kg ha^−1^ (N1) to 210 kg ha^−1^ (N5), the grain N content significantly rose from 69.9 kg ha^−1^ to 131.43 kg ha^−1^ (*p* = 0.001), an 88.03% increase. The N content of the stem and chaff also showed significant growth. In the N5 treatment, the N content of the stem (15.89 kg ha^−1^) and chaff (14.74 kg ha^−1^) were 167.63% and 98.44% higher than those in the N1 treatment, respectively (*p* = 0.002, 0.004). Although the leaf N content significantly increased with the N rate (*p* < 0.001), with no statistically significant benefits observed from high N treatments (N4 and N5), suggesting that leaf N accumulation may become saturated under high N conditions. The total plant N content of wheat increased linearly with the N rate (*p* < 0.001). The total N content in N5 (170.63 kg ha^−1^) was 95.59% higher than in N1 (87.24 kg ha^−1^), with the highest contribution from grain N (77.03%). Notably, when the N rate exceeded 105 kg N ha^−1^ (N3), the N content of grain and the whole plant did not significantly increase further.

**Table 1:** Crop nutrient uptake at wheat maturity (kg ha^−1^).

Nutrient	Treatment	Crop nutrient uptake (kg ha^−1^)				
		Leaf	Stem	Chaff	Grain	Total
N	N1	3.98±0.07d	5.94±0.17c	7.43±0.25c	69.90±0.99c	87.24±0.61c
	N2	5.33±0.15c	9.67±0.23b	10.74±0.17b	111.45±0.5b	137.18±0.19b
	N3	8.18±0.12b	12.57±0.8ab	12.25±1.37ab	128.52±3.71a	161.53±1.46a
	N4	8.69±0.11a	14.51±1.24a	13.21±0.9ab	131.93±6.06a	168.35±6.39a
	N5	8.58±0.21ab	15.89±1.81a	14.74±0.89a	131.43±4.15a	170.63±3.3a
	ANOVA *p*-value	< 0.001	0.002	0.004	0.001	< 0.001
P	N1	0.25±0.01c	0.33±0.03b	0.36±0.07b	7.78±0.42b	8.72±0.39b
	N2	0.24±0.01c	0.55±0.07ab	0.56±0.02a	9.48±0.31ab	10.83±0.28a
	N3	0.38±0.01b	0.56±0.04ab	0.67±0.07a	10.31±0.87a	11.92±0.91a
	N4	0.39±0.02b	0.67±0.02a	0.74±0.06a	10.30±0.77a	12.09±0.72a
	N5	0.49±0.02a	0.76±0.14a	0.63±0.01a	9.65±0.43ab	11.53±0.54a
	ANOVA *p*-value	< 0.001	0.023	0.005	0.075	0.017
K	N1	2.55±0.10cd	20.43±0.53c	5.68±0.44b	7.01±0.59c	35.67±0.31d
	N2	2.11±0.14d	28.91±1.19b	9.27±0.30a	10.03±0.48b	50.31±1.74c
	N3	3.09±0.15b	30.61±1.18b	9.39±0.14a	12.32±0.59a	55.40±0.9b
	N4	3.66±0.18a	34.59±0.31a	10.15±0.14a	10.62±0.68ab	59.01±0.39ab
	N5	3.01±0.21bc	35.85±1.34a	9.65±0.75a	11.02±0.77ab	59.52±1.82a
	ANOVA *p* -value	0.001	< 0.001	< 0.001	0.002	< 0.001

N-fertilizer rates were: N1, non−fertilized control; N2–N5, annual application of 52.5, 105.0, 157.5, and 210.0 kg N ha^−1^, respectively (2003–2022). Values are means ± SD (*n* = 3). Different letters in a column indicate significant differences between treatments (*p* < 0.05).

The N rate significantly affected the P content in wheat leaves, stems, and chaff, but its direct effect on grain P content was weak (**Table 1**). In leaves, the P content in N5 (210 kg ha^−1^) (0.49 kg ha^−1^) was significantly higher than in N1 (0.25 kg ha^−1^), a 95.98% increase (*p* < 0.001). The P content of the stem increased significantly with the N rate, with N5 (0.76 kg ha^−1^) showing a 126.79% increase compared to N1 (0.33 kg ha^−1^) (*p* = 0.023). The P content in the chaff also showed a significant increase, with N5 (0.63 kg ha^−1^) being 77.75% higher than N1 (0.36 kg ha^−1^) (*p* = 0.005). However, only the N3 and N4 treatments significantly increased the grain P content by 32.54% and 32.42% compared to N1, with no statistically significant benefits were observed among N treatments (*p* = 0.075). At the whole−plant level, total P content increased by 24.24-38.74% compared to N1 (8.72 kg ha^−1^; *p* = 0.017), although no statistically significant benefits were observed among N treatments.

The N rate significantly increased the K content in various organs and the K content in wheat (**Table 1**). The response of K accumulation in stems to the N rate was the most significant. The K content in the stems of the N5 treatment (35.85 kg ha^−1^) was 75% higher than that in N1 (20.43 kg ha^−1^) (*p* < 0.001). The grain K content also significantly increased with the N rate, with N5 (11.02 kg ha^−1^) being 57.45% higher than N1 (7.01 kg ha^−1^) (*p* = 0.002). The K content in leaves peaked at 3.66 kg ha^−1^ when the N rate was 157.5 kg ha^−1^ (N4), which was 43.38% higher than in N1, but it slightly decreased to 3.01 kg ha^−1^ in the N5 treatment, indicating that high N input may limit the distribution efficiency of K in leaves. The K content in the chaff significantly increased with the N rate, with N5 (9.65 kg ha^−1^) being 69.92% higher than N1 (5.68 kg ha^−1^) (*p* < 0.001). The total plant K content of wheat showed a significant upward trend with the N rate (p < 0.001), with the K content in the N5 treatment (59.52 kg ha^−1^) being 66.87% higher than in N1 (35.67 kg ha^−1^). Among these, stem K accounted for 64.65% of the total increment, while grain and chaff accounted for 16.81% and 16.65%, respectively. Notably, when the N rate exceeded 157.5 kg ha^−1^ (N4), the increase in K content slowed down, with only a 0.9% increase from N4 to N5.

#### 3.3.2 Nutrient residual in 0−100 cm soil layer

**Figure 6:**
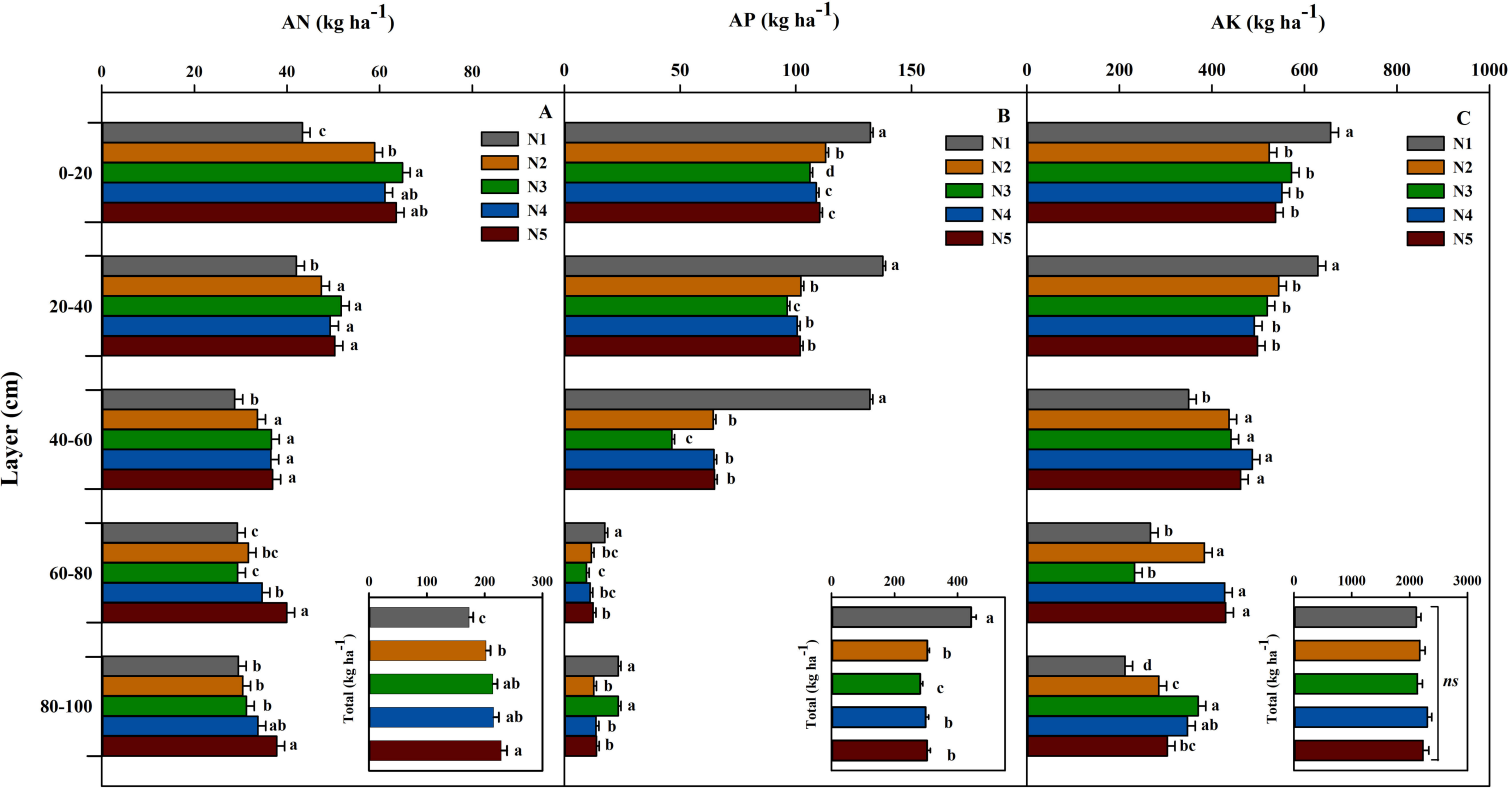
**(A)** Inorganic N residual (AN, kg ha^−1^), **(B)** available P residual (AP, kg ha^−1^), **(C)** available K residual (AK, kg ha^−1^) in the 0−200 cm soil layers at maturity. N-fertilizer rates were: N1, non−fertilized control; N2–N5, annual application of 52.5, 105.0, 157.5, and 210.0 kg N ha^−1^, respectively (2003–2022). Error bars indicate the standard deviation. Bars with different letters indicate significant differences between treatments (*p* < 0.05).

N-fertilizer rate significantly affected the AN residual across all soil layers (**Figure 6A**). In the 0–20 cm soil layer, the AN residual in the N1 treatment (0 kg ha^−1^) was the lowest (43.34 kg ha^−1^), significantly lower than in the N treatments (36.02-49.83%) (*p* < 0.001). As the N rate increased, the AN residual significantly increased, with N3 (105 kg N ha^−1^) reaching the highest value (64.93 kg ha^−1^), which was 49.83% higher than N1. However, when the N rate exceeded 105 kg N ha^−1^, the AN residual in the surface layer did not significantly increase further. In the 20–40 cm and 40–60 cm soil layers, residual AN under N treatments (N2-N5) increased significantly by 12.92-28.39% relative to N1, though no significant differences were observed across the N treatments. In the deeper 60–80 cm and 80–100 cm soil layers, the AN residual in N5 (39.93, 37.80 kg ha^−1^) was significantly higher than in other treatments (*p* = 0.002, 0.015), being 36% and 28% higher than N1, respectively. The total AN residual in the 0–100 cm soil layer increased significantly with the N rate (*p* = 0.003), with the total N residual in N5 (228.51 kg ha^−1^) being 32.20% higher than in N1 (172.85 kg ha^−1^). Notably, the contribution of N in the deep layer (60–100 cm) accounted for 34.05% of the total increment.

The response of N’s vertical distribution to the N rate was asymmetric: N accumulation in the surface layer (0–40 cm) was primarily due to biological uptake, while accumulation in the deep layer (60–100 cm) was mainly due to physical migration. For instance, the AN residual in N5 at 80–100 cm was 28.17% higher than in N1, whereas its residual at 0–20 cm was only 46.72%, much lower than in N3 (49.83%).

The N rate significantly reduced the AP residual in each soil layer (**Figure 6B**). In the 0–20 cm soil layer, the AP residual in N1 was the highest (132.25 kg ha^−1^), significantly higher than in all N treatments (*p* < 0.001), with the residual in N3 (105 kg N ha^−1^) being the lowest (106.06 kg ha^−1^), a decrease of 19.80%. Similarly, the AP residual in the 20–40 cm soil layer also showed a significant downward trend, with N3 having the lowest residual (96.24 kg ha^−1^), a decrease of 30.08%. In the middle soil layers (40–60 cm and 60–80 cm), the N rate significantly reduced the AP residual (*p* < 0.001). In the 40–60 cm soil layer, the residual AP under N1 (132.09 kg ha^−1^) was significantly higher than under the N treatments; for instance, it decreased by 50.91% in N5 (64.84 kg ha^−1^). In the 60–80 cm soil layer, the AP residual in N1 (17.50 kg ha^−1^) was significantly higher than in N3 (9.49 kg ha^−1^, *p* < 0.001), a decrease of 45.80%. Notably, across the N treatments (N2, N4, N5), no significant differences in AP residual were observed within the 40–60 cm layer(*p* > 0.05). The response of AP residual in the deep soil (80–100 cm) to the N rate was different. The available P residual in N1 and N3 (23.25, 23.26 kg ha^−1^) was significantly higher than in other N treatments (for example, the available P residual in N5 was 13.84 kg ha^−1^, *p* < 0.001), with high N input (210 kg ha^−1^) potentially reducing its effectiveness by inhibiting microbial activity or accelerating P fixation. The total AP residual in the 0–100 cm soil layer decreased significantly with the increase in N rate (*p* < 0.001), with the total AP residual in N1 (442.73 kg ha^−1^) being 57.30% higher than in N3 (281.45 kg ha^−1^). Among these, the contribution of AP in the surface layer (0–40 cm) accounted for 41.91% of the total decline, the contribution in the middle layer (40–80 cm) accounted for 58.10%, and the contribution in the deep layer (80–100 cm) accounted for 0%.

The N rate significantly influenced the vertical distribution pattern of AK (**Figure 6C**). In the 0–20 cm and 20–40 cm soil layers, the residual AK under N1 was the highest (657.03 and 629.5 kg ha^−1^, respectively), significantly exceeding that under the other N treatments (*p* = 0.002 and 0.001, corresponding to an increase of 14.83-25.39%). No significant differences in residual AK were observed among the other N treatments. Conversely, the AK residual in deeper soil layers increased significantly with higher N-fertilizer rates. In the 40–60 cm soil layer, the AK residual in the N2-N5 treatments increased by 24.92-39.35% compared to N1. In the 60–80 cm soil layer, the AK residual of N2, N4, and N5 (427.68-429.97 kg ha^−1^) was significantly higher than that of N1 (267.13 kg ha^−1^, *p* < 0.001), with increases of 43.79%, 60.10%, and 60.96%, respectively. The response of AK residual in the 80–100 cm soil layer to N rate was significant (*p* < 0.001), with the highest residual observed in N3 (370.34 kg ha^−1^), an increase of 74.62% compared to N1 (212.09 kg ha^−1^).

In conclusion, the responses of AN, AP, and AK to N-fertilizer rates showed significant differences. AN mainly accumulated in the surface layer, with higher N treatments promoting deeper migration. The AN residual in the 0–100 cm soil layer increased significantly with higher N-fertilizer rates (N5 increased by 32.20% compared to N1, *p* = 0.003). The residual of AP in each soil layer generally decreased with higher N-fertilizer rates, with a significant decrease in the AP pool in the 0–100 cm soil layer (N5 decreased by 31.49% compared to N1, *p* < 0.001), indicating limited deep activation. The residual of AK decreased in the surface layer but increased significantly in the middle and deep layers. The AK pool in the 0–100 cm soil layer remained stable (*p* = 0.519), highlighting the vertical redistribution capability of K.

#### 3.3.3 N balance

Based on the N input and output project of the soil-crop system for spring wheat planting, the N balance of the soil-crop system was evaluated (**Table 2**). When calculating the apparent N balance, the soil layer was defined within the range of 0–100 cm, corresponding to the soil layer accessible to the aboveground part of the wheat. The results showed that the total N input included four components: fertilizer input N, soil inorganic N before sowing (N_min_), soil mineralized N, and N deposited into farmland through wet deposition. Significant differences in N input, output, and surplus were observed under different N rate levels. As the N rate increased (N1-N5), both total N input and output of the system significantly increased.

**Table 2:** N balance in wheat soil system (kg ha^−1^).

Item	N1	N2	N3	N4	N5
A: Total N input	262.59±6.5e	348.96±7.38d	404.85±3.05c	485.51±3.04b	544.66±4.17a
(i) N-fertilizer rates	0	52.5	105	157.5	210
(ii) N_min_	136.08±3.67c	169.95±3.53b	173.34±4.26b	201.5±4.01a	208.15±0.86a
(iii) Mineralization	124.01	124.01	124.01	124.01	124.01
(iv) Wet deposition	2.5	2.5	2.5	2.5	2.5
B: Total N output	265.93±5.74e	355.96±7.38d	411.85±3.05c	492.51±3.04b	551.66±4.17a
(v) Crop uptake	87.24±0.61c	137.18±0.19b	161.53±1.46a	168.35±6.39a	170.63±3.3a
(vi) Soil residual	172.85±7.1c	202.06±7.21b	213.78±7.21ab	215.3±7.21ab	228.51±7.21a
(vii) Apparent loss	5.83±2.03d	16.72±5.53cd	36.54±8.38c	108.86±11.43b	152.52±3.05a
N surplus: (vi) + (vii)	178.68±6.29e	218.79±7.44d	250.32±1.85c	324.16±4.61b	381.03±4.18a
C: N input-output ratio	/	0.38+0.00d	0.65+0.01c	0.94+0.04b	1.23+0.02a

N-fertilizer rates were: N1, non−fertilized control; N2–N5, annual application of 52.5, 105.0, 157.5, and 210.0 kg N ha^−1^, respectively (2003–2022). Values are means ± SD (*n* = 3). Different letters in a column indicate significant differences between treatments (*p* < 0.05).

In terms of N input, chemical N fertilizer (i), pre-sowing soil inorganic N (ii), and mineralized N (iii) were important N sources for the system. With the increase in N rate, the residual of soil inorganic N before sowing significantly increased (from 136.08 kg ha^−1^ for N1 to 208.15 kg ha^−1^ for N5). This increase may be attributed to the previous crops’ insufficient N utilization under high N conditions, resulting in N residual accumulation in the soil.

Regarding N output, crop uptake (v) increased with the N rate, but growth plateaued after the N3 treatment (161.53 kg ha^−1^), with no statistically significant benefits observed from higher application rates (N4 and N5). This indicated that when the N rate exceeded the N3 level (105 kg N ha^−1^), additional N supply did not significantly enhance wheat N uptake, and the agronomic efficiency of N fertilizer decreased. Soil residual N (vi) also varied significantly among treatments, increasing with higher N-fertilizer rates from 172.85 kg ha^−1^ for N1 to 228.51 kg ha^−1^ for N5, demonstrating that high N input led to greater N retention in the soil.

**Figure 7:**
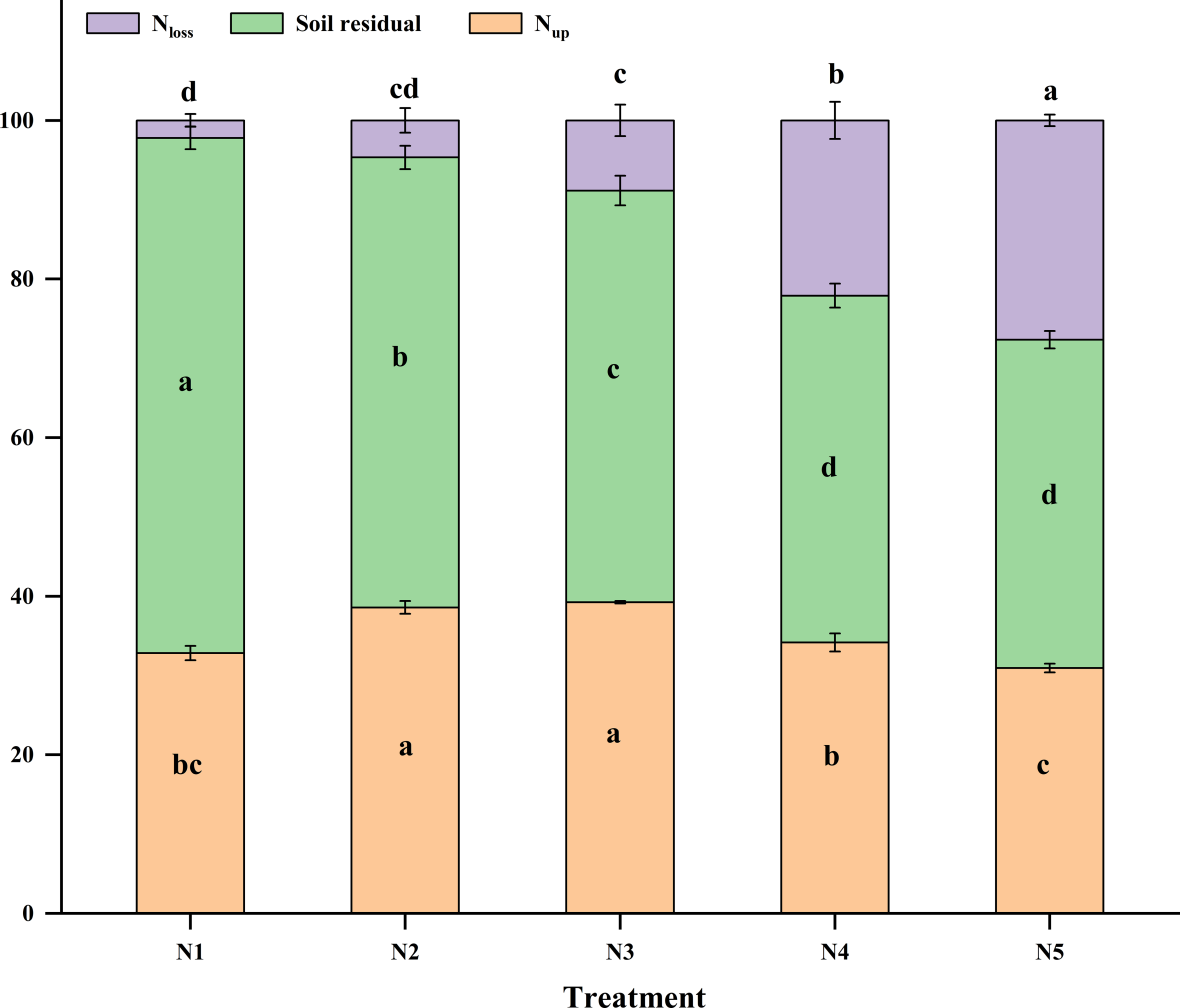
Proportion of N output components. N-fertilizer rates were: N1, non−fertilized control; N2–N5, annual application of 52.5, 105.0, 157.5, and 210.0 kg N ha^−1^, respectively (2003–2022). Error bars indicate the standard deviation. Bars with different letters indicate significant differences between treatments (*p* < 0.05).

The most critical aspect was the apparent N loss (vii), which sharply increased with higher N-fertilizer rates. From N1 to N5, N loss surged from 5.83 kg ha^−1^ to 152.52 kg ha^−1^. Particularly under high N treatments N4 and N5, the losses were as high as 108.86 and 152.52 kg ha^−1^, accounting for 22.1% and 27.6% of the total output, respectively (**Figure 7**). This indicated that excessive N rate significantly increased N loss through volatilization, leaching, denitrification, and other processes, leading to fertilizer waste and substantial environmental risks.

From the perspective of N balance, all treatments exhibited N surpluses, which significantly increased with higher N-fertilizer rates, from 178.68 kg ha^−1^ for N1 to 381.03 kg ha^−1^ for N5. This surplus N, mainly comprising soil residual N and apparent N loss, posed a potential source of environmental pollution. Continuous high N input would lead to excessive saturation of the soil N pool, increasing the risk of N migration to water and the atmosphere. The N input-output ratio also increased significantly from 0.38 in N2 to 1.23 in N5.

In conclusion, an appropriate amount of N fertilizer input (such as N3) can meet crop N demand and maintain a relatively reasonable N balance. However, excessive N rates (such as N4 and N5 levels) do not further improve crop yield or N uptake but significantly increase N loss and soil N surplus, posing a threat to the ecological environment.

The N export pathways during the wheat season primarily include three components: crop uptake, soil residual, and N loss. Different N treatments (N1–N5) significantly impacted the distribution ratios of these three components. Regarding the proportion of crop uptake relative to total output, as the N rate increased, the proportion of crop uptake initially increased and then decreased. The uptake ratios under the N2 and N3 treatments were the highest, at 38.57% and 39.22%, respectively, significantly higher than those of other treatments. The uptake ratio for the N5 treatment was the lowest, at only 30.93%, indicating that excessive N rate is not conducive to the effective transfer of N to crops. The proportion of soil residual N relative to total output decreased significantly with the increase in N rate. The soil residual proportion under the N1 treatment was the highest, reaching 64.95%, whereas for the N4 and N5 treatments, it decreased to 43.72% and 41.41%, respectively. This suggests that high N rate weakens soil N fixation ability, potentially leading to greater N loss. The proportion of N loss increased significantly with higher N-fertilizer rates. The loss ratio for the N1 treatment was only 2.20%, while for the N5 treatment, it surged to 27.66%, more than a tenfold increase. This indicates that an excessive N rate significantly exacerbates N loss through volatilization or leaching, reducing N use efficiency and posing environmental risks. In conclusion, reasonable control of the N rate can enhance crop N uptake and utilization, reduce soil N residual and loss, and is crucial for efficient N use in wheat and environmental protection.

**Figure 8:**
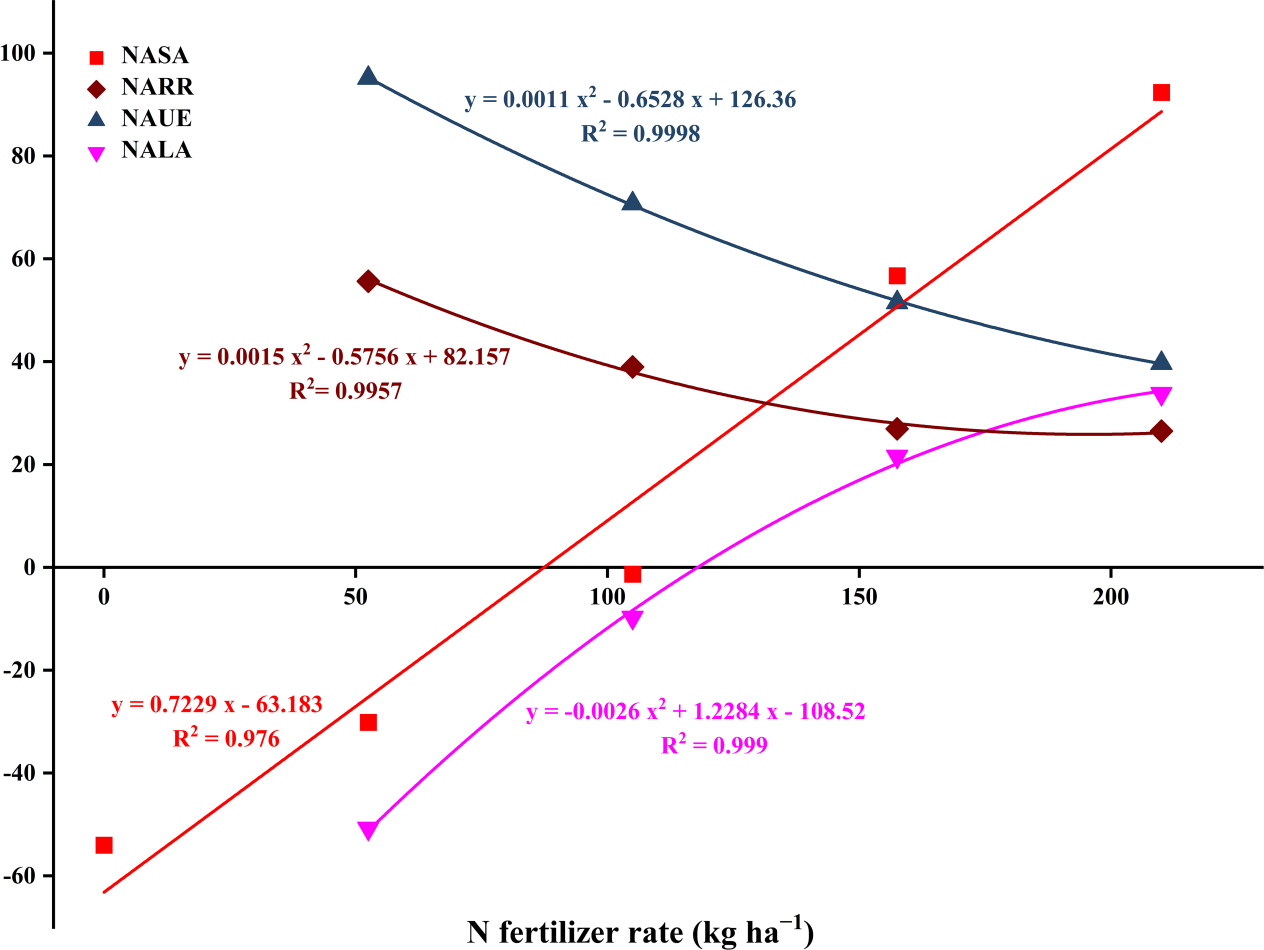
Relation of N fertilization rates and N balance of the wheat-soil system. NASA, N apparent surplus amount; NARR, N apparent residual rate, NAUE, N apparent utilization efficiency; NALA, N apparent loss rate.

The analysis of N use efficiency and soil N surplus and deficit under different N treatments (**Figure 8**) shows that the N rate significantly affects the fate of N fertilizer and its ecological impact. First, regarding N use efficiency, with increasing N-fertilizer rates (N2–N5), the N apparent utilization efficiency (NAUE) significantly decreased, from 95.12% under the N2 treatment to 39.71% under the N5 treatment, a decrease of 58.25%. Simultaneously, the N apparent residual rate (NARR) in the 0–100 cm soil layer also followed this trend, significantly decreasing from 55.65% under N2 to 26.50% under N5, with reductions of 42.75%, 106.48%, and 109.95% under N3–N5 compared to N2, respectively. The simultaneous decline of these two indicators shows that an excessive N rate not only fails to enhance crop uptake of fertilizer N but also reduces the preservation ratio of N within the soil-crop system, leading to fertilizer resource waste.

Secondly, regarding N loss and environmental risk, the N apparent loss rate (NALA) significantly increased with higher N-fertilizer rates (*p* < 0.001). In the low N (N2, -50.76%) and medium N (N3, -9.73%) treatments, the loss rate was negative, indicating a net consumption of the soil background N pool and a low environmental loss risk. However, when the N rate increased to N4 (157.5 kg ha^−1^) and N5 (210 kg ha^−1^), the loss rate turned positive, reaching 21.55% and 33.79%, respectively. This indicates that under high N input, more than 20% of fertilizer N is neither absorbed by crops nor retained in the soil, but directly enters the environment, posing a significant pollution risk. According to the fitting equation, when the N rate was 117.6 kg ha^−1^, NALA was zero.

The N apparent surplus amount (NASA) gradually shifted from a significant deficit to a significant surplus with increased N-fertilizer rates, ranging from -44.01 kg ha^−1^ in the N1 treatment to 102.34 kg ha^−1^ in the N5 treatment, an increase of 19.98-108.95%. The N surplus and deficit in the N3 treatment was approximately 8.69 kg ha^−1^, close to zero equilibrium, indicating that N input and output were roughly balanced, and the system was relatively stable. However, the N4 and N5 treatments exhibited a substantial N surplus, consistent with the large amounts of N residual and losses shown in **Table 2**, further confirming that high N input leads to excessive saturation of the soil N pool. According to the fitting equation, when the N rate is 87.4 kg ha^−1^, NASA is 0 kg ha^−1^, which may be near the balance point between crop demand and environmental capacity. Beyond this threshold, NASA will rapidly accumulate, potentially causing environmental issues such as groundwater pollution or greenhouse gas emissions.

In summary, although the utilization efficiency of a low N rate (such as N2) is high with low loss rates, it may deplete the soil N pool, which is not conducive to sustainable agricultural production. Conversely, excessive N rates (such as N4 and N5) ensure crop uptake and soil residual in the current season but at the cost of extremely low utilization efficiency and high environmental losses. The N3 treatment demonstrated a relatively ideal balance point in this study, maintaining high N use efficiency and keeping the soil N budget nearly balanced, making it the recommended N rate to achieve coordination between wheat production and ecological environment protection.

**Figure 9:**
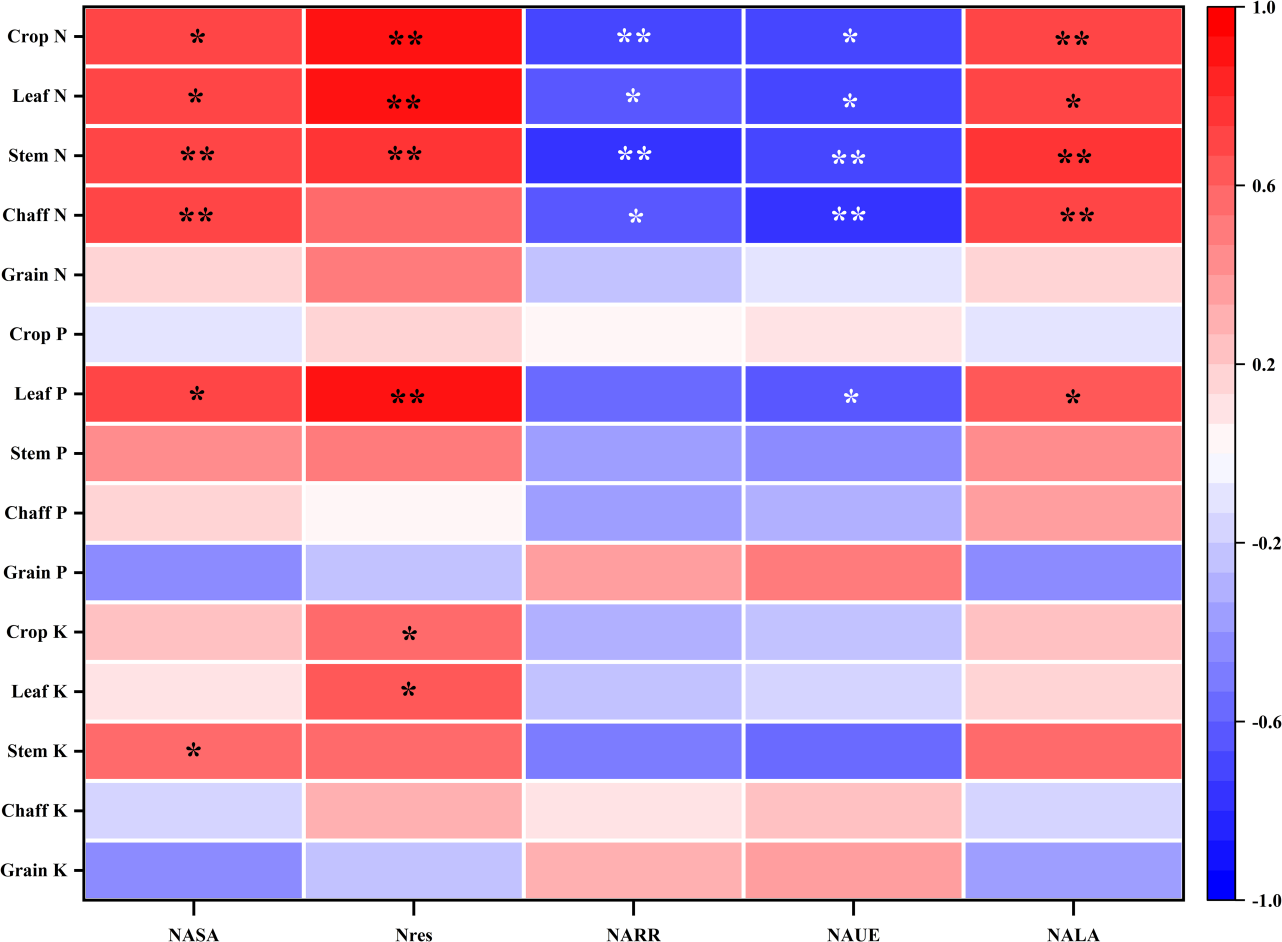
Relationship between N balance and nutrient concentration (g kg^−1^). * *p* < 0.05; ** *p* < 0.01.

Correlation analysis of nutrient indices (N, P, K) and N balance parameters (NASA, N_res_, NARR, NAUE, NALA) in different plant parts showed significant differences (**Figure 9**). There was a significant or extremely significant positive correlation between NASA and N concentrations in all organs (except grains) of mature plants. This confirmed that soil N surplus directly promotes the excessive uptake and luxury accumulation of N in crop vegetative organs. However, this promotion effect was not significantly reflected in grain N concentration, indicating that excessive N mainly promotes vegetative growth rather than effectively transforming into grain protein. Soil inorganic N accumulation (N_res_) also showed a highly significant positive correlation with N concentration in most organs, especially with leaf N concentration as high as 0.932**. This clearly demonstrates that residual inorganic N in soil under high N-fertilizer rates is the direct source promoting increased N concentration in crop plants, particularly in leaves.

From the perspective of N-use efficiency, NAUE and NARR were generally significantly negatively correlated with N concentration in various vegetative organs, while NALA was significantly positively correlated with them. This series of close correlations reveals a key mechanism: when the N rate is too high, N use efficiency decreases, and more N is not effectively used by crops or safely retained in the soil but is lost to the environment. At the same time, the N concentration in the vegetative organs of crops increases abnormally. This shows that an increase in N concentration in vegetative organs is not a sign of high N use efficiency but a symptom of excessive N, low N use efficiency, and increased loss.

Furthermore, the analysis of P and K concentrations revealed that their correlation with N indicators was significantly lower than that of N concentration. Most correlations were not statistically significant. An exception was found where leaf P was significantly positively correlated with NASA and N_res_, suggesting that high N environments may synergistically enhance P accumulation in leaves. However, no significant correlations were observed between P and K concentrations in grains and N indicators.

In summary, the correlation analysis statistically confirmed that i indiscriminate increases in N application to elevate N concentration in crops, particularly in vegetative organs, results in reduced N use efficiency, increased N loss rates, and a substantial surplus of soil N. This management strategy fails to effectively improve grain quality and exacerbates the risk of N pollution to the environment.

## 4 Discussion

### 4.1 Regulation of N rate on nutrient distribution in wheat

Nitrogen (N) application rate not only determine N nutrition in wheat but also acts as a “master switch” regulating nutrient distribution pattern within the plant (Dordas, 2009). This study results demonstrated that increasing N application significantly enhanced the concentration and accumulation of both N and K in wheat organs (**Figure 2**, **Table 1**), highlighting a strong synergistic effect of N on the uptake of these two nutrients. This synergistics relationship can be attributed to several mechanisms: (i) as a key component of chlorophyll and proteins, N enhances photosynthetic capacity and overall plant growth, thereby increasing biomass accumulation and demand for K, which function as a major osmotic regulator and enzyme activator (Gaju et al., 2014); (ii) The uptake and assimilation of N (especially NO_3_) require K ions to maintain electrical neutrality and cellular charge balance (Aziz et al., 2025; Hafsi et al., 2007); and (iii) N fertilizer promotes root growth and development, thereby expanding the soil volume explored by roots and indirectly enhancing K uptake (Wang et al., 2024; Zörb et al., 2014).

In contrast to the synergistic relationship observed between N and K, P distribution exhibited unique antagonistic and competitive characteristics. Although P accumulation in leaves (increased by 53.89-95.98% except for N2 treatment), stems (increased by 63.15-100.10%), and chaff (increased by 56.90-107.23%) increased with higher N application rates, grain P concentration decreased significantly under higher N application rate. Specifically, the N5 treatment resulted in a 17.80% reduction in grain P concentration (2.27 g kg⁻¹). Path analysis further confirmed that the N-fertilizer rate indirectly exerted a negative effect on grain yield by reducing grain P concentration (-0.606). This finding supports the previous hypothesis that excessive N-induced biomass accumulation leads to a “dilution effect” of P in grains. Because P is a critical component of energy metabolism (ATP) and genetic material (DNA and RNA), reduced grain P concentration may constrain grain filling capacity and ultimately limit yield formation (Malhotra et al., 2018). In this study, the N5 treatment resulted in the highest P concentration in leaves at maturity (0.63 g/kg) and the lowest P concentration in grains (2.27 g/kg), providing strong evidence for restricted P translocation to reproductive organs under excessive N supply. Several mechanisms may explain this phenomenon. (i) Biomass dilution: rapid vegetative growth induced by high N supply can outpace P translocation to grains, resulting in lower grain P concentration. (ii) Energy competition: N assimilation in plants (such as nitrate reduction and ammonium assimilation) is an energy-intensive process. Under high N conditions, plants may allocate more photo assimilates (ATP and reducing power) to N metabolism, thereby reducing the energy available for P transport to grains (such as P ester synthesis and P transporter activation) (Kant et al., 2011). (iii)rhizosphere processes: the N fertilization (especially ammonium N) may cause acidification in the rhizosphere microenvironment (Sun et al., 2020; Khan et al., 2025), affecting the form and availability of soil P. However, since sufficient P fertilizer was applied in all treatments in this study, internal plant transport rather than soil P availability is likely the dominant factor governing the observed decline in grain P concentration.

The positive correlation between N and yield across all wheat organs underscores its essential role as a “life element” (**Figure 2**. Conversely, the negative correlation between grain P concentration and yield, compared to the positive correlation between chaff P concentration and yield, implies that P content in the chaff, serving as the “final channel” for nutrient transport to the grain, may reflect the efficiency of P transport. Therefore, an optimal N rate (such as N3) should ensure adequate N supply without obstructing the P transport pathway to the grain, thereby achieving a balance of high yield and superior grain P nutrition.

### 4.2 Vertical differentiation of soil nutrients

Our study demonstrated depth-specific and nutrient-specific effects of N rates on soil nutrients. Residual available N in the 0–100 cm soil layer increased significantly with higher N rates (Wang et al., 2019). Notably, under high N treatments (N4 and N5), the available N residual in deeper soil (60–100 cm) increased significantly, accounting for 34.05% of the total increment. This indicated that when the N rate exceeded crop demand and the surface soil’s holding capacity, N (primarily in the form of highly mobile NO_3_) began to leach into deeper soil layers. This finding aligned with previous studies (Liu et al., 2025a; Sun et al., 2024), who identified high N inputs as a primary cause of nitrate leaching and groundwater pollution in the North China Plain. The N3 treatment (64.93 kg ha^−1^) exhibited the highest available N residual in the surface layer (0–20 cm), whereas the N5 treatment (average 38.87 kg ha^−1^) showed higher available N residual in the deep layer (60–100 cm). This suggests that an N rate of 105 kg N ha^−1^ retains more N in the root-dense area for crop uptake, whereas 210 kg N ha^−1^ primarily promotes ineffective N migration below the root zone.

Contrary to the characteristics of N migration and accumulation, the response of the soil available P pool to N rates exhibited an overall depletion (**Figure 5B**). Under the N5 treatment (210 kg N ha^−1^), the available P residual in the 0–100 cm soil layer decreased by 31.49% compared to N1. This phenomenon can be attributed to several mechanisms: (i) Enhanced chemical fixation: Nitrification following N rate (especially ammonium N) can cause localized acidification, leading to the formation of more insoluble calcium phosphate in calcareous soils, thus reducing P availability (Liu et al., 2025c; Xu et al., 2022); (ii) Biological fixation competition: High N levels stimulate microbial activity, potentially increasing microbial fixation of available P, making it less accessible to crops in the current season (Yang et al., 2024); (iii) Changes in root morphology (Xin et al., 2021): High N may inhibit root growth, limiting crop access to P in deeper soil layers. Our results serve as a critical warning: In dryland wheat systems, long-term excessive N application not only leads to N loss (apparent loss 16.72-152.52 kg ha^−1^) but also exacerbates the depletion of the soil P pool, creating new and more challenging nutrient limitations in the future and threatening the sustainability of the agricultural system (Zhang et al., 2015).

The response of soil K demonstrated a distinct pattern of vertical redistribution (**Figure 5C**). The available K in the surface layer (0–40 cm) decreased, while it significantly increased in the deeper layer (40–100 cm). This phenomenon may be attributed to several factors: (i) Crop uptake: crops absorb a substantial amount of K from the surface soil. (ii) Downward leaching: as NO_3_⁻ leaches downward, K^+^, as a companion ion, may also migrate downward (Römheld and Kirkby, 2010). (iii) N fertilization affects root distribution, potentially promoting crops to absorb and utilize more K from the middle soil layers. Although the total K pool in the 0–100 cm soil layer remained stable (*p* > 0.05), this redistribution from the active surface layer of the root system to the deeper layers may increase the difficulty of K acquisition for crops during critical growth periods.

### 4.3 Critical transition of N balance

The N input-output ratio increased from 0.38 in N2 to 1.23 in N5, with the N surplus ranging from 178.68 to 381.03 kg ha^−1^ (**Table 2**), indicating a shift from N deficiency to N surplus. A high N surplus suggests not only resource wastage but also potential environmental risks (Cui et al., 2018). Our findings are consistent with previous studies (Cassman et al., 2002), who observed that when the N input-output ratio exceeds 0.8, system sustainability significantly decreases in similar ecological regions. Notably, the N3 treatment (105 kg N ha^−1^) maintained relatively low N loss (36.54 kg ha^−1^) and moderate N surplus (250.32 kg ha^−1^) while ensuring full crop uptake, reflecting better ecological and economic benefits.

The N balance analysis in this study provided a robust tool for quantitatively evaluating the agronomic benefits and ecological risks associated with different N fertilization strategies (Mogollón et al., 2018). N apparent utilization efficiency (NAUE) significantly declined from 95.12% in N2 to 39.71% in N5 (**Figure 8**), consistent with the globally observed “diminishing returns of N fertilizer” (Zhang et al., 2015). Notably, the N apparent loss rate (NALA) was 0% when the N rate was approximately 117.38 kg ha^−1^. Under N4 (157 kg N ha^−1^) and N5 (210 kg N ha^−1^) treatments, NALA shifted from negative to positive, reaching 21.55% and 33.79%, respectively. This marks a fundamental transition from sustainable consumption of the soil background N pool to active N emission pollution (Chen et al., 2014).

The equation fitting for N apparent surplus amount (NASA) indicated that at an N rate of 87.4 kg ha^−1^, the system achieved a theoretical N budget balance (NASA = 0 kg ha^−1^). However, our study demonstrated that the NASA of the N3 treatment (105 kg N ha^−1^) was 8.69 kg ha^−1^, which was closest to the equilibrium point, with grain N, K accumulation, and whole plant N concentration reaching their plateau stages. This suggests that 105 kg ha^−1^ is the optimal balance point for crop demand and environmental capacity under the experimental conditions. Below this value (e.g., N1, N2), although the risk of loss is low, it may lead to the depletion of the soil N pool (NASA of N1 is -44.01 kg ha^−1^) and is unsustainable. Above this value (e.g., N4, N5), it results in a substantial N surplus (NASA of N5 is 102.34 kg ha^−1^), sacrificing N use efficiency and increasing environmental losses. The inverse relationship between loss rate and residue rate, as shown by the synchronous decrease in N apparent residual rate (NARR) (55.65% to 26.50%) and increase in NALA (-50.76% to -33.79%), indicates that the soil N fixation capacity tends to be saturated under high N input. Consequently, unused N is lost in large quantities through physical or biological processes, posing a potential threat to the ecosystem (Ma et al., 2008; Yan et al., 2025). The marginal effect of N accumulation between the N4 and N5 treatments, where the increase in NASA (56.68 to 92.34 kg ha^−1^) was significantly higher than the decrease in NARR (26.95% to 26.50%), further supports that environmental loss is the main driver of N surplus (Xia et al., 2017).

Correlation analysis (**Figure 9**) statistically revealed the “symptoms” of high N management: the increase in N concentration in vegetative organs was closely related to the decrease in NAUE and NALA. This finding challenges the traditional notion that “the greener the plant, the better,” clearly indicating that N luxury accumulation in vegetative organs is not a sign of high yield and efficiency but an early warning signal of improper N management, low utilization efficiency, and increased environmental risk (Cameron et al., 2013; Uwiragiye et al., 2024).

In conclusion, we recommend an optimal N rate of 105 kg N ha^−1^ for spring wheat in semi-arid, rain-fed areas. This rate meets crop requirements and ensures high levels of grain N (128.52 kg ha^−1^) and K accumulation (12.32 kg ha^−1^). The system balance was maintained, keeping the soil N budget near equilibrium with an output-input ratio of 0.65, which prevents soil N pool depletion (NARR, 38.98%) or excessive surplus (NASA, 8.69 kg ha^−1^). Additionally, the N loss risk (NALA, -9.73%) was kept at a low level. This approach aligns with the principle of precision fertilization as advocated in China’s “zero fertilizer growth” action plan (Cui et al., 2018). Future N management should shift from solely pursuing yield to the coordinated regulation of crop demand, soil N supply capacity, and environmental capacity (Liu et al., 2013; Wang et al., 2018).

However, this study has identified several scientific issues that require further investigation. First, the molecular physiological mechanisms by which high N rate inhibits P transport to grains remain unclear. Future studies should integrate transcriptomics and metabolomics to investigate the gene expression and regulatory networks of key transporters under the interaction of N and P. Second, the N balance calculated in this study is an apparent calculation and does not differentiate the specific contributions of various loss pathways (e.g., leaching, volatilization, N_2_O emission). Future research should quantify these different loss fluxes and their environmental footprints using techniques such as ^15^N isotope tracing. Furthermore, this study did not evaluate soil microbial processes or soil health indicators. Excessive N inputs may substantially alter soil microbial community structure, enzyme activities, and organic matter turnover, all of which are critical for regulating nutrient cycling and nutrient use efficiency. The absence of these assessments may have led to an underestimation of the broader ecological consequences associated with high N application rates.

## 5 Conclusion

An optimal nitrogen (N) fertilizer rate of ≤105 kg N ha^−1^ significantly increased the concentration and accumulation of N and K in various wheat organs, promoting efficient nutrient translocation to the grains. In contrast, excessive N application (>157.5 kg ha^−1^) led to overaccumulation of N and K in vegetative tissues (stems and leaves) and inhibited P transport to grains, resulting in a lower grain P concentration and reduced nutrient use efficiency. While N fertilization increased the soil N pool, it also elevated the risk of N migration into deeper soil layers. The available P pool decreased under high N inputs, suggesting that P could become a new limiting factor, Whereas K showed clear redistribution trend from surface soil toward deeper horizons. When N application exceeded approximately 117.6 kg ha^−1^, the apparent N loss rate became positive, highlighting increased environmental risks. The highest N treatment (N5) resulted in a substantial N surplus (102.34 kg ha^−1^), posing a potential threat of groundwater quality and greenhouse gas emissions. Considering crop uptake, fertilizer use efficiency, and environmental sustainability, an N rate of 105 kg N ha^−1^ is recommended for spring wheat in dryland regions. This rate achieves an optimal balance between yield, nutrient utilization, and environmental protection, providing a scientific basis for sustainable N management in semi-arid, rain-fed agroecosystems.

## Data Availability

The original contributions presented in the study are included in the article/supplementary material. Further inquiries can be directed to the corresponding author.
